# The Influence of Micro-Organisms in Producing Caries of the Teeth

**Published:** 1884-12

**Authors:** Arthur S. Underwood

**Affiliations:** Lecturer on Dental Anatomy and Physiology at the Dental Hospital of London Medical School, etc.


ARTICLE VIII.

### ON THE INFLUENCE OF MICRO-ORGANISMS IN THE PRODUCTION OF CARIES.—CONTINUED.

BY ARTHUR S. UNDERWOOD, M. R. C. S., AND L. D. S., ENG.,

Lecturer on Dental Anatomy and Physiology at the Dental Hospital of London Medical School, etc.

[Read before the Odontological Society of Great Britian, April 7, 1884.]

The next experiment we shall describe is a repetition of one described by Dr. Miller, with this extension, that it has been carried out under septic and aseptic conditions. Two 1-oz. bottles were half filled with saliva; to this was added bread in both cases, and in one carbolic acid; into these were introduced fragments of healthy teeth extracted for regulation and split. The saliva was the same—*i. e.*, from the same mouth—and taken at the same time, the bread from the same piece, the teeth were halves of the same tooth; both fluids were neutral. The bottle not containing carbolic speedily became the scene of an active growth of micro-organisms, for the greater part micrococci; a thick felt of this growth formed at the surface of the fluid. At the end of three months the surfaces of the teeth in the impure flask had become softened, but not discolored; the surface of those in the pure flask was unaltered, the fluid in the pure flask was neutral, that in the impure one strongly acid. Thus in the impure flasks acids were generated as well as germs, or, as we think seems more rationally to express it, by the germs. The teeth from these fluids are here for your inspection. Lastly, a number of teeth are here for your inspection in which a change has certainly taken place after a long immersion in septic fluids.

It is plain, then, that whereas in the absence of germs no change takes place that could be supposed to be caries, and that in the presence of germs a change does take place, which, although it would render our views more cut and dried to call caries, we are bound to confess is a very weak caries if it be caries at all; and further, remembering that unmistakable caries does affect natural teeth or artificial which have no connection with the vascular system, we are led to inquire whether organisms, that are undoubtedly the prime factors in the production of other diseases, afford us any analogy of a loss of power when placed in strange conditions. The answer to this inquiry is very plain and direct, and completely in the affirmative.

Pasteur, Naegali, and other equally reliable authorities, have discovered that special organisms alter their character when grown under unusual conditions. Even though they continue to grow and reproduce their species, their special properties weaken and die out in the unnatural surroundings. Thus, to take a couple of typical instances, Pasteur, by growing bacillus anthracis at a peculiar temperature, "attenuated" the organism to such an extent that he was able to vaccinate animals with it—that is, he rendered this special bacillus, which, in its normal condition, would, if inoculated into an animal, produce anthrax, practically inocuous by changing its surrounding conditions. Again, Naegali has been able so to modify the properties of a lactic acid producing bacterium that it produced an ammoniacal fermentation. Lastly, certain pathogenic bacteria require a special soil in which to grow and a special temperature, otherwise the growth ceases altogether. Moreover, the presence of a number of extraneous organisms does in some cases hinder the active growth of others. A similar difficulty has attended the attempt to grow the organisms of gonorrheal ophthalmia, and such instances may be almost indefinitely multiplied; the conclusion is, however, pretty plain, namely, that special organisms require special surroundings to display their special activity, and we are inclined to think that in the case of the micro-organism of caries a living mucous membrane is one of those conditions.

In view of these facts, we feel ourselves justified in claiming to have established as a permanent addition to dental pathology the fact that micro organisms of a special form do play an important part—nay, more, that they are an essential factor in the production of caries. We think many of these experiments show that these organisms do much of the work—at any rate, as far as the removal of the limesalts goes—by the aid of an acid which they produce. We feel convinced that the process, to be effective, must be carried on in a living mouth, probably because that is the only situation in which the special germs are really active. Analogy seems to warrant this deduction; but, before concluding, we wish to impress upon the meeting, and through the meeting upon all who take an interest in the matter, that all we claim for the germs is, that without them and their products the process could not go on at all. The essential element that was not present in our artificial experiments is present in the mouth; it is possible, and we consider from the analogies quoted above, *probable*, that this important element is the living buccal mucous membrane as a scene of operations, and the constant succession of the proper germs. Enough has, however, been laid before you to induce us to hope that this Society will indorse the statement made by us in the August of 1881, and repeated more fully now, that micro-organisms play an important part in the production of caries, and that the dental pathology of the future must include them among the most powerful factors in the production of this disease.

We have only, in conclusion, to thank you, gentlemen, for your kind attention, and to leave the question in your hands for discussion.—*New Eng. Journal of Dentistry.*

